# Lateral double magnetic tunnel junction device with orthogonal polarizer for high-performance magnetoresistive memory

**DOI:** 10.1038/s41598-022-24075-y

**Published:** 2022-11-17

**Authors:** Stanislav Sin, Saeroonter Oh

**Affiliations:** Department of Electrical and Electronic Engineering, Hanyang University, Ansan, 15588 Republic of Korea

**Keywords:** Engineering, Electrical and electronic engineering

## Abstract

Magnetic tunnel junction (MTJ)-based memory devices have larger switching delay and energy consumption, compared to cache or dynamic random access memory. In order to broaden the applications of the magnetoresistive random access memory, reducing the switching time and energy consumption of the MTJ is required. Here, a novel lateral double MTJ with an orthogonal polarizer is proposed. The proposed device consists of three ferromagnetic regions: the first pinned region (PR1) with perpendicular magnetic anisotropy (PMA), a free region (FR) with PMA, and the second pinned region (PR2) with in-plane magnetic anisotropy (IMA). PR1 and PR2 are placed on top of the oxide barrier, which separates them from the FR, comprising a lateral double MTJ structure. The current pulse through PR2 helps to perturb the magnetization of the FR. Since the angle between PR2 and FR is 90°, the initial torque increases significantly, decreasing switching delay by 4.02 times and energy-delay product by 7.23 times. It is also shown, that the area of the access transistor can be reduced by approximately 10%, while maintaining the same energy-delay product and reducing gate RC delay.

## Introduction

Among the current non-volatile memory candidates, magnetoresistive random access memory (MRAM) enjoys high endurance, long retention time, and compatibility with the CMOS process^1–11^. After two decades of extensive research, magnetic tunnel junction-based (MTJ) MRAM has been successfully commercialized^4,12^. A conventional MTJ (CMTJ) device consists of a pinned layer (PL), an oxide barrier, and a free layer (FL). If magnetization of the FL is antiparallel to that of the PL, the resistance of MTJ is high, and low otherwise.

In conventional spin-transfer torque-driven (STT) MRAM, FL is switched by a spin-polarized electrical current. The switching power and delay of such MRAM are still one order of magnitude higher compared to its silicon counterpart, namely SRAM.

In the next-generation MRAM–spin-orbit torque (SOT) MRAM, a more efficient way to inject spin current using the spin Hall effect in heavy metals was utilized, which led to decreased power consumption^6,12–17^. However, it is impossible to exert out-of-plane torque using the conventional spin Hall effect. Hence, SOT MRAM needs the assistance of an external magnetic field in order to switch the FL with perpendicular magnetic anisotropy (PMA). Field-free switching can be realized with the help of STT or geometrical symmetry breaking^13,14^. Recent studies demonstrate that perpendicularly polarized spin current can be injected using unconventional SOT effects^18–21^, but such effects are immature and need extensive research. Another drawback of such memory is a low spin Hall angle $\theta _{SHE}$, which will be a problem in nanometer-scale MTJs, as will be explained below. The spin Hall polarization is given as^6,13,13–15^:

1 $$\begin{aligned} P_{SHE}=\frac{I_s}{I_c}=\frac{A_{FL}}{W_{HM}t_{HM}}\theta _{SHE}(1-sech(t_{HM}/\lambda _{sf})) \end{aligned}$$

where $I_s$ and $I_c$ are the spin and charge currents, respectively, $A_{FL}$ is the area of FL; $W_{HM}$ and $t_{HM}$ are the width and thickness of the heavy metal, respectively; $\lambda _{sf}$ is the spin-flip length of a heavy metal.

As can be seen from Eq. (1), polarization efficiency decreases with the area of FL. Considering circular MTJ with a diameter of 5 nm, $\lambda _{sf} = 1$ nm, and a typical value of $\theta _{SHE} = 0.3$, the maximal value of $P_{SHE}$ is 0.45. Comparing it with the typical polarization efficiency of STT ($P_{STT}\approx 0.5{-}0.6$)^5,17^, it can be concluded that at scaled dimensions, SOT MRAM tends to have a larger switching current compared to the STT MRAM. Therefore, SOT MRAM will also require larger access transistor, and as a consequence, larger cell area.

The main reason of the long switching delay in STT/SOT MRAM (particularly when the current is lower than a threshold value) is the low STT/SOT when magnetization is collinear^22,23^. It is known that STT is proportional to the vector cross product ${\textbf{m}}_p\times {\textbf{m}}$^2,3,5–9,12^, where ${\textbf{m}}_p$ and ${\textbf{m}}$ are the normalized magnetizations of PL and FL, respectively. Therefore, when the polar angle between ${\textbf{m}}$ and ${\textbf{m}}_p$ is $\theta = \{0, \pi \}$, STT vanishes. Consequently, the switching of the conventional MTJ highly relies on the random thermal fluctuations of the magnetization vector. Such switching is referred to as “thermally-assisted switching.” It requires high current densities to be applied for a long time. When ${\textbf{m}}$ is collinear to ${\textbf{m}}_p$, a large amount of energy is dissipated before the magnetization starts to barely move. It is reasonable to think that STT is maximal at $\theta = \pi /2$; however, the asymmetry term $\varepsilon$ slightly shifts the torque maxima from $\pi /2$ towards the antiparallel state.

In order to resolve these issues, several novel spintronic devices with orthogonal polarizers were proposed, such as STT MRAM with perpendicular polarizer^2,3,24–26^, and complementary polarizer spin-transfer torque (CP-STT) MRAM^8^. In prior works^24–26^, a perpendicular polarizer was deposited on top of a conventional in-plane magnetic anisotropy (IMA) MTJ to exert additional torque, which caused a magnetization precession. The device was switched by an alternating current pulse, and sub-nanosecond switching was achieved. However, the device switching was non-deterministic and required precise pulse-width control. In other works^2,3^, the IMA polarizer was added to PMA MTJ, which resulted in one order of magnitude improvement in the switching time. Fong et al.^8^ reported a device with two antiparallel PLs and depending on the magnetization of the FL, the current path was chosen to perform magnetization switching from an antiparallel state, which decreased switching time and power. But since the initial magnetization of the FL was also collinear, the starting torque was low.

In conventional two-terminal MTJs with orthogonal polarizer, current flows through the entire structure, introducing additional heating and losses. Note also that after magnetization reaches the equator, in-plane torque will interfere with the switching process. Here, a novel lateral double MTJ (LDMTJ) device with an orthogonal polarizer is proposed. Unlike the previous designs, the proposed device has both PMA and IMA PLs patterned on the oxide barrier. Thanks to the lateral double MTJ structure, additional flexibility in terms of current magnitude and pulse widths through PMA and IMA is gained. Hence, in-plane torque can be selectively injected into the FR. A CoFeB/MgO/CoFeB LDMTJ device was simulated in this study. The distribution of switching time, mean energy, and the energy-delay product was obtained through a large number of simulations. The effects of thermal fluctuations and non-uniform currents were included in the micromagnetic model. We find the LDMTJ device has $\sim$ 10% smaller access transistor’s area, for the same value of energy-delay product and reduced gate RC delay.

## Results

### Proposed lateral double MTJ with orthogonal polarizer

**Figure 1:**
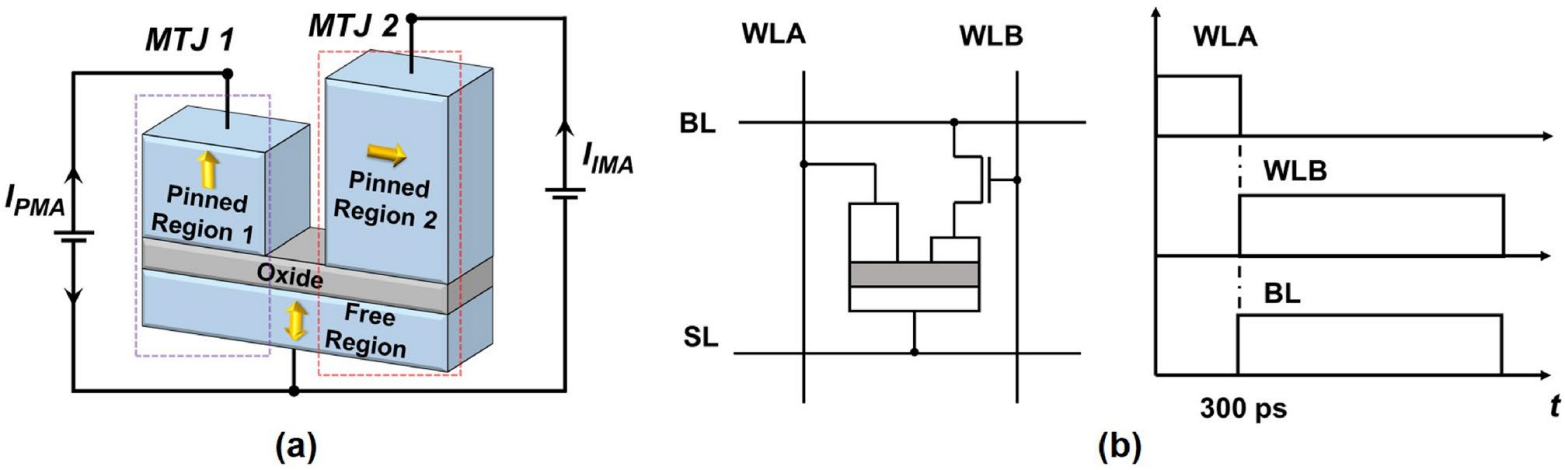
(**a**) Structure of the proposed device. Free region and pinned region 1 have PMA, whereas the pinned region 2 has IMA. (**b**) Proposed 1T1LDMTJ MRAM cell and its WRITE operation.

The proposed LDMTJ device with the orthogonal polarizer is shown in Fig. 1a. The bottom layer comprises free CoFeB ferromagnet with PMA and is named the free region (FR). A thin MgO barrier and thick ferromagnetic layer are deposited on the FR. Two rectangular pinned regions have length $L_{PR1} = L_{PR2} = 18$ nm, width $W_{PR1}=W_{PR2} = 40$ nm, and separation distance of 4 nm. Fabrication of such a structure is undoubtedly challenging with current technologies. Although fabrication details are beyond the scope of this study, we expect with advances in extreme ultraviolet lithography and multiple patterning, such geometry would be more feasible to fabricate. The first pinned region (PR1) is thin, and since the perpendicular anisotropy field is inversely proportional to the thickness^1^ it has PMA. The thickness of the second pinned region (PR2) is greater than the critical value. Hence, the demagnetizing field dominates over perpendicular anisotropy, resulting in IMA where the energy minimum lies along the width direction. The device dimensions and simulation parameters used in this study are listed in Table 1. Contact leads are connected to each ferromagnet, and the entire device can be viewed as a lateral double MTJ with a shared FR. The first MTJ comprises FR and PR1, it is used for the READ operation, and finishing the WRITE operation. The second MTJ comprises FR and PR2, and used only during the initial phase of the WRITE operation, in order to decrease incubation time. Magnetostatic interaction from PR2 was neglected, presuming its effect on READ operation is small. Additionally, it can be compensated by a synthetic antiferromagnet stack^27,28^.

The proposed 1T1LDMTJ memory cell and its WRITE operation are depicted in Fig. 1b. The source line (SL) is connected to a FR, and one access transistor is connected between PR1 and bitline (BL). The cell has two wordlines: WLA is connected to a PR2, and WLB is connected to a gate of the transistor. WLA is activated first. It feeds a relatively short current pulse $I_{IMA}$ through the MTJ2 in order to perturb the initial magnetization state. Subsequently, BL and WLB are clocked simultaneously in order to feed the current $I_{PMA}$ through MTJ1. The signal from BL creates the required voltage difference across MTJ1, while WLB opens an access transistor. The short delay of WLB is optional, and WLB can be activated simultaneously with WLA. Simple-model simulations of LDMTJ predict a minor performance change when WLA and WLB pulses overlap. However, simultaneous injection of spin currents with different polarization may lead to a complicated spin-scattering inside the ferromagnet. Therefore, the actual impact of sequential and simultaneous gating should be experimentally verified. In this study, sequential gating of WLA and WLB was considered for the sake of generality. Taking this into account, the choice of the pulse overlap is left to the circuit engineer.

Due to the MTJ2, exerted torque is relatively large since the angle $\theta = \pi /2$ initially. Hence, for efficient current utilization the first pulse should flow through MTJ2 to decrease the incubation period, and the second pulse should be applied afterward to finish the switching.

### Simulation results

In order to verify the effectiveness of the LDMTJ, it was compared with CMTJ by numerical simulations. Both devices have identical FRs with the same dimensions. The device dimensions and corresponding simulation parameters are listed in Table 1. The pulse-width of $I_{IMA}$ was 0.3 ns. It corresponds to a clock frequency of 3.33 GHz, which is achievable by high-performance CMOS circuits^29^.

**Table 1:** Simulation parameters^30^ used for performance evaluation.

Parameter	Description	Value
*L*	Length of the free region	40 nm
*W*	Width of the free region	40 nm
*d*	Thickness of the free region	1 nm
$d_{ox}$	Thickness of the oxide barrier	1.1 nm
$K_i$	Interfacial anisotropy energy coefficient	0.32 $\text {mJ/m}^2$
$M_s$	Saturation magnetization	0.625 × $10^6$ A/m
$\alpha$	Damping coefficient	0.05
*A*	Exchange energy coefficient	13 × $10^{-12}$ J/m
$\Lambda _{FR}, \; \; \; \Lambda _{PR}$	Phenomenological asymmetry coefficients	1.1, 1.6
$P_{FR} = P_{PR}$	Polarization efficiency	0.58
$\Delta$	Thermal stability	40
$\tau _{IMA}$	Pulse-width of the current through MTJ2	0.3 ns
$RA_p$	Resistance-area product in parallel state	10 $\; \Omega \; \upmu \text {m}^2$

For a fair comparison, it was assumed that CMTJ and LDMTJ have identical FRs with the same dimensions.

**Figure 2:**
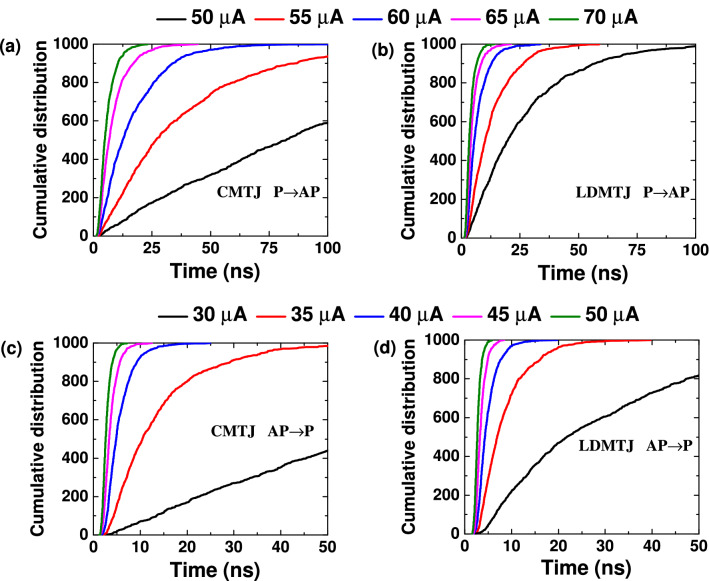
Cumulative distribution of the switching time for (**a**) conventional and (**b**) lateral double MTJ devices switched from parallel to antiparallel state; (**c**) conventional and (**d**) lateral double MTJ devices switched from antiparallel to parallel state.

It is worth noting that switching of an MTJ is a stochastic process. To obtain meaningful statistical data, a large number of simulations with different current values were performed. The distribution of the overall time, required for switching of CMTJ and LDMTJ versus switching current is shown in Fig. 2. For the LDMTJ device $I_{IMA} = I_{PMA}$ condition was considered. Each curve was built using 1000 points (20,000 simulations were performed in total). As can be seen, the switching time obeys Boltzmann distribution and this fact is consistent with previous studies^22,23^. Switching from parallel to antiparallel state (P $\rightarrow$ AP) is shown in Fig. 2a,b, for CMTJ and LDMTJ, respectively. The proposed device demonstrates a shorter incubation time and steeper distribution. Figure 2c,d show switching from antiparallel to parallel state (AP $\rightarrow$ P) for CMTJ and LDMTJ, respectively. A similar picture can be observed at currents below 50 μA, but at higher currents, CMTJ eventually becomes slightly faster.

**Figure 3:**
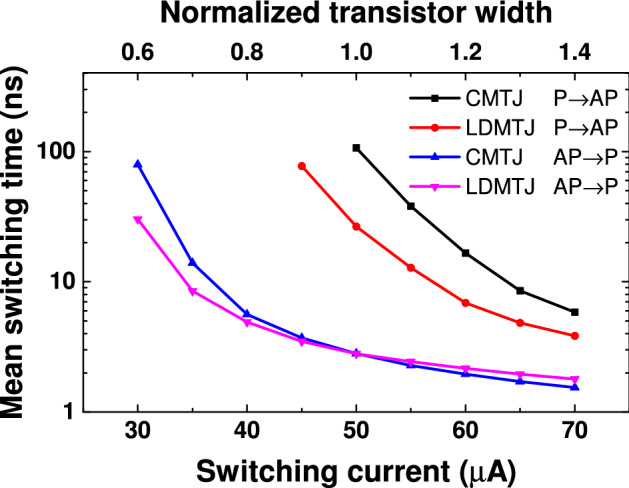
Mean switching time of the CMTJ and LDMTJ devices. Each point on the plot is averaged over 1000 simulations. Top axis shows the effective transistor width normalized to the width required to maintain the current of 50 μA.

The dependence of the mean switching time on the switching current for CMTJ and LDMTJ is shown in Fig. 3. The top axis shows the effective transistor width normalized to the width required to maintain the current of 50 μA. It is the lowest current required for P $\rightarrow$ AP switching of the CMTJ. Due to the shorter incubation time, the LDMTJ device is especially effective at lower currents. As the current rises, the role of the incubation time decreases as with the performance gain of LDMTJ. The maximum performance enhancement for the P $\rightarrow$ AP case is demonstrated at the lowest switching current of 50 μA, where the mean switching time is reduced by 4.02 times from 106.80 ns to 26.59 ns. For the AP $\rightarrow$ P case, the mean switching time is reduced from 79.46 ns to 30.28 ns by 2.62 times. Interestingly, this figure shows that at currents above 50 μA, the switching time of the CMTJ from AP $\rightarrow$ P is slightly shorter, compared to that of LDMTJ. At 70 μA, CMTJ with a mean time of 1.54 ns was 1.16 times faster than LDMTJ with a mean time of 1.79 ns. The figure also suggests that the width of the access transistor can be decreased by $\sim$ 12% while maintaining the same P $\rightarrow$ AP switching delay.

As was demonstrated in the previous section, though the proposed LDMTJ device is much faster at low currents, its benefits in AP $\rightarrow$ P switching vanish at $I > I_{cr}$. There are two reasons for that:

1. STT from PMA magnet becomes more effective, compared to that from IMA magnet. It is known that STT from IMA magnet is more effective at current densities lower than the critical value $J < J_{cr}$, and much higher than that $J \gg J_{cr}$^26^.
2. Note that in the proposed device, only a part of the device is subject to a current flow at a time. LDMTJ requires more time to switch the domains to the right side of PR1, compared to CMTJ, where current flows uniformly through the whole FR. At lower currents, this drawback is mitigated by a decreased incubation time, but as the current rises its role becomes less significant, and exchange interaction becomes crucial.

We suggest that the first issue can be alleviated by optimization of $I_{IMA}$ magnitude and pulse duration. But the apparent trade-off between switching speed and active power, together with the limit on the critical voltage of the MTJ will complicate the design procedure. The second issue can be mitigated as the MTJ dimensions are decreased. In this study, the exchange constant was $A = 13 \cdot 10^{-12} \; \text {J/}\text {m}^\text {2}$. It corresponds to an exchange length $l_{ex}=\sqrt{(2A/\mu _0 M_s^2 )} = 7.28$ nm. If the length of LDMTJ is decreased so that the inactive part of FR will be smaller than $l_{ex}$, magnetization vector will move uniformly in the whole region due to the strong exchange interaction.

**Figure 4:**
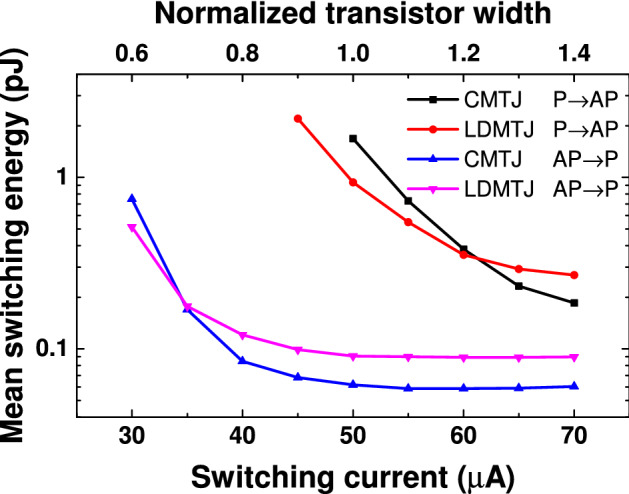
Mean switching energy of the CMTJ and LDMTJ devices. Each point on the plot is averaged over 1000 simulations.Top axis shows the effective transistor width normalized to the width required to maintain the current of 50 μA.

The comparison of mean active switching energies of CMTJ and LDMTJ is shown in Fig. 4. Two energy terms were considered in the loss calculations, namely magnetic energy due to the magnetization reversal and energy due to Joule heating. Magnetic losses were on the order of $10^{-27}$ J, whereas Joule losses were on the order of $10^{-12}$ J. Hence, the magnetic energy can be neglected. According to Fig. 4, LDMTJ tends to be more energy demanding for moderate to high currents. The reason is the increased resistance of MTJ1 and MTJ2. Noting that the resistance is proportional to the area, and that areas of MTJ1 and MTJ2 are almost half of FR’s area, it can be concluded that LDMTJ’s power increases roughly by 4 times. The difference in active energy is lower than the difference in power, since resistance of MTJ2 is lower than resistance of MTJ1 in antiparallel state.

**Figure 5:**
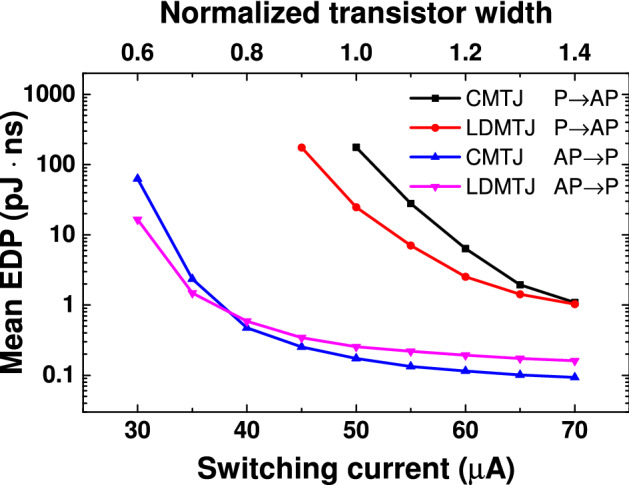
Mean energy-delay product of the CMTJ and LDMTJ devices. Each point on the plot is averaged over 1000 simulations. Top axis shows the effective transistor width normalized to the width required to maintain the current of 50 μA.

Mean energy-delay product (EDP) comparison is given in Fig. 5. Although LDMTJ utilize more energy compared to the CMTJ, its EDP can still be less than that of CMTJ. For P $\rightarrow$ AP switching at 50 μA, EDP was decreased 7.23 times from 180.06 to 24.92 pJ ns, but the EDP at 70 μA is almost equal for both devices: 1.08 pJ ns for CMTJ and 1.06 pJ ns for LDMTJ. For AP $\rightarrow$ P switching, LDMTJ’s EDP at 30 μA was reduced by 3.81 times: from 59.49 to 15.59 pJ ns. However, due to the increased power, the EDP reduction effect of the LDMTJ over the CMTJ for AP $\rightarrow$ P switching is lost for currents over 40 μA. Despite this, LDMTJ still has lower EDP averaged between P $\rightarrow$ AP and AP $\rightarrow$ P switching.

## Discussion

As was demonstrated in the previous section, the proposed LDMTJ device is much faster at low currents. The important consequence of this is the shrinkage of access transistor size. It can be seen from Fig. 5 that LDMTJ requires less current for the same EDP. For example, CMTJ at 55 μA has an EDP of 27.97 pJ ns for P $\rightarrow$ AP switching, and 0.13 pJ ns for AP $\rightarrow$ P switching. Hence, the average EDP of CMTJ is 14.05 pJ ns. Meanwhile, LDMTJ at 50 μA has EDPs of 24.92 and 0.25 pJ ns for P $\rightarrow$ AP and AP $\rightarrow$ P switching, respectively. The average EDP of LDMTJ is 12.59 pJ ns. Consequently, the area of access transistor can be decreased by approximately 10%, while still retaining switching speed and energy efficiency. Note that a smaller transistor area leads to shorter transistor delay due to the decreased gate capacitance.

In summary, a novel LDMTJ device with orthogonal polarizer was proposed. The bottom CoFeB FR has PMA and is shared between two MTJs. Top CoFeB PRs have different magnetizations owing to the different thicknesses. Because the initial current pulse flows through the PR2 with IMA, the incubation time reduces significantly. Simulation results demonstrated that the mean energy-delay product could be decreased by 7.23 and 3.81 times for P $\rightarrow$ AP and AP $\rightarrow$ P switching, respectively, at low currents. Alternatively, the EDP gain can be sacrificed for a decreased area of the access transistor. At dimensions less than $l_{ex}= 7.28$ nm, the proposed device could achieve better performance gain for scaled MRAM applications.

## Methods

The micromagnetic simulations of CMTJ and LDMTJ were performed in MATLAB software. In order to consider non-uniformities of current distribution and magnetization, the device was divided into 20 domains along the length. Magnetization dynamics was calculated by the well-known phenomenological Landau-Lifshitz-Gilbert (LLG) equation:

2 $$\begin{aligned} \frac{d\mathbf {m_{i}}}{dt}(1+\alpha ^2) = -\gamma _0 \left [\mathbf {m_{i}} \times ({\textbf{H}}_{eff,i} - \frac{\hbar J \beta }{2q\mu _0 M_s d}{\textbf{m}}_{p,i}) + \alpha \mathbf {m_{i}} \times (\mathbf {m_{i}} \times ({\textbf{H}}_{eff,i} - \frac{\hbar J \varepsilon _{i}}{2q\mu _0 M_s d}{\textbf{m}}_{p,i}) ) \right ] \end{aligned}$$

where ${\textbf{m}}$ is the unit-vector of the magnetization; $\alpha$ is the phenomenological damping coefficient; $\gamma _0$ is the gyromagnetic ratio, ${\textbf{H}}_{eff}$ is the effective magnetic field due to the different contributions, namely ${\textbf{H}}_{ani}$ is the perpendicular anisotropy field, ${\textbf{H}}_{exc}$ is the exchange field, ${\textbf{H}}_{d}$ is the demagnetizing field, and ${\textbf{H}}_{th}$ is the random thermal field; $\hbar$ is the reduced Planck’s constant; *J* is the current density; $\varepsilon$ is the asymmetry term; *q* is the elementary charge; $\mu _0$ is the permittivity of vacuum; $M_s$ is the saturation magnetization; *d* is the thickness of FR; ${\textbf{m}}_p$ is the magnetization of the pinned region (either PR1 or PR2). It was assumed that magnetostatic interactions were negligibly small or could be minimized by a proper technique^27,28^.

Contributions of the effective magnetic field were calculated as follows^31^:

3 $$\begin{aligned} {\textbf{H}}_{ani, \; i}= & {} \frac{2 K_i}{d \mu _0 M_s} \hat{{\textbf{m}}}_z \cdot {\textbf{m}}_i, \end{aligned}$$

4 $$\begin{aligned} {\textbf{H}}_{d, \; i}= & {} \sum _j N_{i-j} {\textbf{m}}_j M_s, \end{aligned}$$

5 $$\begin{aligned} {\textbf{H}}_{exc, \; i}= & {} \frac{2 A}{\mu _0 M_s} \nabla ^2 {\textbf{m}}_i, \end{aligned}$$

6 $$\begin{aligned} {\textbf{H}}_{th, \; i}= & {} \sqrt{\frac{2k_B T \alpha }{\gamma _0 \mu _0 M_s V_{FR} \Delta t}} \mathbf {\sigma } \end{aligned}$$

where $K_i$ is interfacial anisotropy coefficient, $\hat{{\textbf{m}}}_z$ is the unit vector along *z*-axis, *N* is the demagnetizing tensor, $k_B$ is the Boltzmann constant, *T* is the temperature, $V_{FR}$ is the volume of FR, $\Delta t$ time discretization step, and $\mathbf {\sigma } = [\sigma _x \; \; \sigma _y \; \; \sigma _z]$ are the Gaussian distributed random numbers. The interfacial anisotropy constant $K_i$ was calculated in order to satisfy a thermal stability factor of 40$k_B T$, required to maintain a 10-years retention period at 300K. It is difficult to evaluate device stability in the case of a multidomain structure. Therefore, the thermal stability factor was calculated using an expression for single domain^1,30^:

7 $$\begin{aligned} \Delta = \frac{E_b}{k_B T} = \frac{(K_i/d -0.5 M_s^2[N_z-N_y]) V_{FR}}{k_B T} \end{aligned}$$

note that the equation above is given in SI units. Mean initial angle can be calculated based on $\Delta$ as:

8 $$\begin{aligned} \theta _0 = \sqrt{1/2\Delta } \end{aligned}$$

The asymmetry term is calculated by the following equation^32^:

9 $$\begin{aligned} \varepsilon = \frac{q_+}{A_+ + A_- ({\textbf{m}} \cdot {\textbf{m}}_p)} + \frac{q_-}{A_+ - A_- ({\textbf{m}} \cdot {\textbf{m}}_p)} \end{aligned}$$

10 $$\begin{aligned} q_{\pm } = P_{PR}\Lambda _{PR}^2 \sqrt{\frac{\Lambda _{FR}^2 + 1}{\Lambda _{PR}^2 + 1}} \pm P_{FR}\Lambda _{FR}^2 \sqrt{\frac{\Lambda _{PR}^2 + 1}{\Lambda _{FR}^2 + 1}} \end{aligned}$$

11 $$\begin{aligned} A_{\pm } = \sqrt{(\Lambda _{PR}^2 \pm 1) \, (\Lambda _{FR}^2 \pm 1)} \end{aligned}$$

where $P_{PR}$ and $P_{FR}$ are spin polarization efficiencies in pinned and free regions, respectively; $\Lambda _{PR}$ and $\Lambda _{FR}$ are asymmetry parameters.

The device was divided into 20 domains along the length. LLG for each magnetic domain was solved with the canonical 4th order Runge-Kutta method. For simplicity, $I_{IMA} = I_{PMA}$ was considered, and the duration of the first pulse $\tau _{IMA}$ was set to 300 ps. The second pulse was maintained until the device is fully switched, meaning that $m_z$ reaches -0.95 of its initial value. Non-uniform current distribution was considered assuming that electrons tunnel straightly in *z*-direction. In other words, out-of-plane torque from PR1 affected right 45% of the device, whereas in-plane torque from PR2 affected left 45% of the device. We can make this approximation since

1. The tunneling in an angled direction is much less probable because the electron traveling distance is larger.
2. Spin current is mainly absorbed at the interface of the magnet.
3. Free layer is thin, and the transport can be assumed ballistic.

The aspect ratio of PR1 and PR2 may be varied, however, in this study both areas are assumed to be 45% of the total area. The remaining gap was presumed to be filled with an insulator in order to avoid a short circuit.

The resistance of the MTJ was calculated as follows^30^:

12 $$\begin{aligned} R = R_p \frac{1 + (V/V_h)^2 + TMR}{1 + (V/V_h)^2 + TMR( 0.5 \, (1 + cos {\theta }))} \end{aligned}$$

where $R_p$ is the resistances in a parallel state, *TMR* is the tunneling magnetoresistance ratio at 0 bias, *V* is the bias voltage, and $V_h$ is the voltage at which TMR halves.

The distribution curves were obtained by Monte Carlo simulations. Each curve in Fig. 2 was built using 1000 simulations. Mean values in Figs. 3, 4, 5 were averaged on the same number of simulations.

## Data Availability

The data that support the findings of this study are available from the corresponding author upon reasonable request.
